# The Dentist and the Child

**Published:** 1911-09-15

**Authors:** B. H. Johnson

**Affiliations:** Portland, Tenn.


### THE DENTIST AND THE CHILD.

BY B. H. JOHNSON, PORTLAND, TENN.

Read before the Tennessee Dental Association, May 23, 1911.

We are now reaching the age, in which preventive dentistry is advocated; and in which the old adage that "an ounce of prevention is worth more than a pound of cure," is recognized as being more applicable to dentistry, than any other branch of the great work, of assisting nature to keep soul and body together and in condition to do life's work, and especially to do justice to ones meals. We are fast learning that the whole body is affected largely by the condition of the teeth. Then nature does not provide for the restoration of lost tooth structure, as is often the case with other structures of the body. Therefore every effort should be exerted to prevent their loss.

As the sins of the fathers are visited upon their sons, to the third and fourth generation, so are the defective mouths and teeth of fathers and mothers visited upon their children.

The earliest we can begin prophylaxis is not soon enough. But if the end of the world doesn't come too soon, we shall make a great improvement. By the systematic and determined way the different dental associations are working to get the people educated to the importance and necessity of caring for their teeth, and thereby getting their co-operation, and assistance in caring for their own and their childrens teeth, we are greatly encouraged to hope for perfect teeth some time in the future.

We find most children prejudiced against the dentist, for which there may be good reasons, which we shall need to investigate.

We fail to realize that a childs mind is more impressive than that of the adult, for a child has every thing to learn, even the names and uses of the most common-place things; and as little as we think, it is wonderful to note the rapidity and accuracy of the minds of most children from the age of two to seven. Impressions made at this age seldom loose their influence. A wise Catholic priest once said, "Give me a child until it is seven and it will remain a Catholic the remainder of its life."

Children hear all the magnified horrors of various visits to a dental office, and never forget them. And the dentist is painted in their imaginations as a huge cruel monster, who delights in torturing people, especially women and children.

Most people, especially women, when telling of their experiences in the dental chair, are like the Baptist brother who was prompted by his conscience, and his pastor to make a confession to the church for excessive exaggerating, or as some call it, indulging in hyperbole. He rose with tears in his eyes and said: "Sisters and brethren, I am exceedingly sorry for this great fault of mine, and am determined to break myself of this habit, and ask the prayers of my beloved sisters and brethren for I am sure I have shed barrels and barrels of tears about this one sin."

Parents and nurses often threaten to punish the fretful child with a visit to the horrid old dentist, with the awful forceps and instruments to inflict the severest pains, and the enmity and fear is often so thoroughly instilled in some children in this way, that it not only lasts through childhood, but like other sins it is handed on to their children's children.

These, gentlemen, are the problems with which we have to contend. How shall we go about it? It is my opinion that we will have to make an attack from every conceivable point.

One method is to put forth every effort to eliminate all pain and unpleasantness possible without sacrificing the thoroughness of the work, and learn to be patient with nervous patients. Try to estimate the endurance of every patient and be careful not to overtax him. We should not become so much interested in the operation and its completion as to forget that our patient has feelings and a limit to his endurance. By such tactics we can deminish the scary tales and gain the friendship and recommendation of parents and older sisters and brothers. Then we can more easily get at the child.

We must win the child's confidence and love; this is generally easy if one loves children, if not, it may be impossible in most instances. For it seems that their instinct is very accute, and they watch you much closer than will a grown person, until by your actions you have relieved their little minds of all fear.

I make it a rule to take advantage of every opportunity to gain the affection of every little child in my town and they will talk about you to their little playmates and tell about you giving them some fruit, or a glass of lemonade at the fount, and then they are generally proud to get an opportunity to visit you, and often come alone. I have several little patients under eight years of age, that come up often and ask me if their teeth need any attention. Children seldom forget you if you have relieved them of pain without punishing them. I remember relieving a little patient only four years of age, and her parents afterward moved to another town, and over a year afterward her teeth needed attention, and nothing would do her, but her father take her to the dentist that fixed her other teeth. She was proud to inform me that they had tried to hire her with candies and money and other things to let a dentist at home fix her teeth, but she reckoned she knew who her dentist was. It is best not to have over sympathetic parents with them or in the operating room as they often ask questions that scare the child. Of course, all children can't be handled alike, sometimes we are compelled to be firm but never unkind.

Tell them while working what pretty teeth they will have and how nice it will be to have a little tooth brush and learn to brush their teeth at least twice a day.

Try never to let one go away fretted by you, for they will be hard to bring back.

Parents often ask us the cause of the early decay of childrens teeth. Miller demonstrated that dental caries is due to a number of causes, but the principal and basal one is the growth of oral bacteria. If it were possible to find a child or a number of children, born of parents who had been absolutely temperate in all things from youth up, and had lived in accordance with the true law of continence, would there be any defects in their teeth, or any other physical disabilities?

Dr. John Cowan, of New York, says not. If such be the case then immorality, intemperance and licentiousness may be the primary cause of diseased teeth as well as all other ailments of the body, except those caused by traumatisms.

It is said that there is no other law connected with the directing and governing of the human body and soul that so affects the individuals welfare, happiness and success in life as does the law of continence. For this reason it deserves all the thought and argument that can be brought to bear upon it, especially by those who have taken for their life's work caring for any part of the earthly tabernacles of men's souls.

Milton said—

So dear to heaven is saintly chastity,
That, when a soul is found sincerely so,
A thousand, liveried Angels lackey her.

If these things be true, and we can't deny that they are to a great extent, and we are really trying to save men's teeth, and the people by teaching them to take care of their teeth. Should we not set the example and teach the people that there must be a reformation before they can have perfect health and perfect teeth for their children?

### DISCUSSION.

Dr. N. J. Morrison: The most difficult and objectionable feature of a child's practice is the fillings. You can put five fillings in an adult mouth while you are giving the child a ride on the elevator, pumping your chair up and down. You can have the adult patient finished and the money spent while you are fooling with a child, and yet if you would present a larger bill for the care of the child's teeth than you do for the adult's, you loose the family. Now it is an easy matter to discuss this in our meetings, but not so easy to work it out in the office. On our program I notice somebody is going to offer a plan for raising money to establish a fund for dentists who have to be buried in the pauper's field, and yet the idea of talking fees ever since I can remember has been tabood, you must not talk about your fees, you must not think about your fees. You are not going to get any fee—so much for the fee question.

Now as to the manipulation of the child, my experience has been that if you can get to the mother or the father, especially the mother, and get the mother to instill into the child's mind what a nice place it is to go to the dentist's, how many nice things he has to show him and how he is going to ride you up and down on that elevator chair, and so on, if you can get the mother to do that before she brings the child to the office, it will relieve you of a great deal of nervous strain. Now each individual has his way of handling the child when it comes to the office. It depends altogether on the age of the child, the disposition of the child, the kind of child it is, as to how you are going to get along handling the little fellow. He comes in all scared to death, the best thing if you can do it is to take your chair and get out by the window and take him on your knee and talk to him about something else, the dogs or the cats—there are a thousand ways in which to interest children, and if you can, examine him there on your knee and get his confidence. The thing is, how are you going to manage him to do this. You can't do it by rule, but will have to accept every chance that the occasion offers.

Dr. L. G. Noel: I think the greatest trouble we have with children is falsifying to them, just down-right lying, tell a child you are not going to hurt him, and then just almost murder him. You will never get another chance at a child so treated. I have in mind one little girl, I have been extracting teeth for her, she is losing her deciduous and erupting her bicuspids, they are all in, she has been coming to my office each Saturday morning for the last seven weeks and I take out a tooth each week. She has been coming there with a great deal of anticipated pleasure. She has been bringing two or three of her little playmates with her. Last Saturday, one of them was badly broken down, and the bicuspid tooth was pushed out to one side and crushed down between it and another tooth, and I was perfectly frank with her, and I said, "this one is going to hurt you, it will not hurt you much, but it will hurt you some." I didn't have any trouble at all because she knew what was coming. Now I said next Saturday we will get the other one. It has been my experience that we must be just as truthful with children as with adults. Their parents lie to them too when they come to the dentist. I am confident it is better to be truthful with the child because we want to do his practice the rest of his life, and the best way to get his confidence is to be perfectly truthful with him.

Dr. S. B. Cook: There is one thing that we all neglect, and that is in not getting these children early enough. I have never had any trouble with children when I got them before they had any pain from toothache. I believe we ought to advise the parents first, and especially the mother, and impress them with the idea that the child ought to go to the dentist before it has experienced any pain. If you will do this, I think you will have very little trouble. I have an illustration of that in my own family. I have four children, and three of them never had the toothache, but the last boy did, and I can hardly do anything with him. I want also to emphasize the statement that we should never deceive children. They will believe everything you say until you have deceived them, and after that they will believe absolutely nothing you tell them. The first request

I make of a mother is, "Don't you come with this child." If it is not necessary for you to come, we don't want you present at all. I manage it a great deal better without them. I don't think anybody is any more enthusiastic in regard to oxyphosphate of copper cement than I am. You can talk as much as you please about different materials, but I don't believe there ever has been so good a material made for children's teeth. I have put it into cavities that I could scarcely clean out at all, and been agreeably surprised at the results. I want to say that much because I know there are a great many dentists who do not know about it, or who do not use it.

Dr. S. R. Jones: Frequently a child is introduced to a dentist on the street, in some such speech as this, "Don't you know Dr. Jones, why he is the man that pulls out your teeth?" That is the way we are often introduced. We ought to disabuse the children's minds of anything of that kind as much as possible. Instruct the parents of these children not to refer to us in any such terms. In handling children I think we ought, of course, to be honest with them, and their parents ought to be fair minded and prudent when they speak of us in the child's presence. I often appeal to them in this way, now this will hurt you a little bit, but you can't afford to have your beautiful eyes, pretty cheeks and beautiful dresses that your parents buy for you all spoiled by ugly and irregular teeth that will surely result if these first teeth of yours are not handled right by your dentist. I can often handle children and win their confidence in that way, but the main thing I wanted to stress was the importance of the parents dealing truthfully with the child in respect to the dentist.

Dr. I. O. Templeton: I like the copper cement, I have used it when I didn't know what else to do and got fine results from it, and I want to say that down at Winchester, there are so many children that you can't tell who their mothers are, and a great many of the mothers never see you, I know I have never seen them. A while back a public school teacher asked me to come over and see him. So I went over there, and when I got there I said, "I have come to give you public school teachers a good dose of dentistry." I prepared models from my own childrens mouths, from the smallest one on up, and I displayed those models and talked to the little fellows, and they became interested. It was not a week until I had a case of toothache, the little fellow said, you are the doctor who was talking to us about our teeth, I have the toothache and I thought I would come over here and see what you could do for me. That little fellow started his mother, and the mother came the next day and said, "Charlie told me that you were over at the public school the other day, and I thought I would come over and get you to fix my teeth." I don't know the number of little fellows who have been to me since, talking about their teeth and what to do for them. I think if we are to save the children of the country it has got to be done through the public school teacher, if we get the public school teacher's interest they will teach the children and the children will interest their parents and we will reach the majority of them after a while.

Dr. Walter G. Hutchinson: It is all right to talk about getting the child's confidence, and the mother started to teaching the child, but if there is one man more scared than the child, that man is the dentist himself. I have never seen one yet who, when he saw a child step into his office, that didn't loose his confidence that quick.

Dr. Leonard: A method that has given me a great deal of satisfaction in the treatment of children's teeth, because it eliminates a great deal of pain, is pressure anesthesia. I do not think there is any class of cases that lends itself to this method better than exposed pulps in children's teeth. It is just a question of the application of the cocain to the exposed pulp and it is easily anesthetized by a little pressure. The application will be entirely painless and the patient has no sensation of pain at all. With a sharp burr in the engine you can remove the bulbous part of the pulp from the pulp chamber itself, and from the openings to the canals, not trying to remove it from the roots entirely. These teeth can then be filled immediately and without any probability of having trouble afterwards. I have been using this method for about ten years in that class of cases.

Dr. King: I didn't expect to say anything, but it seems that you have all come to the conclusion that the dentist is an habitual liar when dealing with children; but from what has been said it does not seem that the dentist is the only liar, because the parents seem to be just as untruthful as the dentist, because the parents invariably tell the child that it is not going to hurt. We will take you down to the dentist and have this tooth extracted, and it won't hurt you at all, but when the child comes into the office and you do hurt him, it is all off with you and that child. A short time ago, a lady brought in a little boy about six years old who had an abscessed lowar molar, she was a Christian Scientist, and she had instilled into the child the belief that it would not hurt him at all, and she had got him to the point that he was so confident that it would not hurt him that he came to the office to have the tooth extracted. He sat in the chair and his mother kept telling him to remember that it was not going to hurt him at all. He had the tooth extracted and it hurt the little fellow dreadfully. He indignantly told his mother "to go to hell with her Christian Science, that it did hurt like the devil." That is the way it goes with children, there is only one way, both the dentist and the parent must be truthful with the child. Just as Dr. Morrison said, if you leave the parent at home you can generally manage the child very well. As some one else has said, we can't get them too early, the earlier we get them the better time we shall have, and they will grow up to have confidence in us and we will save more of their teeth.

Dr. B. H. Johnson: I want to thank the gentlemen for both the commendations and condemnations of my paper. As for the advertising any particular brand of cement I do not care to do this, as every one will select the particular brand of cement that works best for him.

As to the fee, I did not mean to say that we should not think of it in children's work, but the point is, we should put our minds on the benefits to the patient, instead of considering our finances so much. The artist, so far as that is concerned, can do his best work when he can leave all pecuniary interests out of the question. We should not think about our own interests primarily, but about the work we are attempting to do. As to the mother's or father's influence on a child, there are very few parents that are not more timid than the children when they get into the chair, unless you have gained their confidence. I think false statements to children are among the gravest offences that we can commit, because they remember longer than grown people, and they remember them after they get older, and it is the quickest way to offend the child and make it impossible for us to gain its confidence and affection. As for getting at the children in school, I think it is one of the very best ways. I was notified by the teacher in our town to give a course of lectures to the school, and we offered prizes for the twelve best papers. The children took a great deal of interest in it, and it soon became of interest on the public streets, and a great deal of interest in good teeth was worked up. Unless the pulps are much inflamed—some times when they are a little bit inflamed—you can apply the oxyphosphate of copper directly to the exposed pulp.

In removing the pulps of children's teeth, unless they are partially dead or much inflamed, they should be kept alive, as it is probable that removing the pulps may retard absorption of the roots, I do not know that it will, but I have noticed that teeth that have been devitalized, when they are extracted that the roots are not absorbed as well as those whose roots are kept alive.

